# Combining plasma biomarkers, clinical parameters, and neuroimaging features for differential diagnosis of Parkinson’s disease and atypical parkinsonian syndromes: a multidimensional modeling approach

**DOI:** 10.3389/fnagi.2026.1727812

**Published:** 2026-01-16

**Authors:** Jian Yao, Jiajia Ma, Peng Li, Xianglian Liao, Jie Zan, Liangshan Hu, Guihua Li

**Affiliations:** 1 Department of Neurology, The Affiliated Guangdong Second Provincial General Hospital of Jinan University, Guangzhou, Guangdong, China; 2 The Second School of Clinical Medicine, Southern Medical University, Guangzhou, Guangdong, China; 3 Department of Laboratory Medicine, The Affiliated Guangdong Second Provincial General Hospital of Jinan University, Guangzhou, Guangdong, China; 4 School of Clinical Medicine, Jinan University, Guangzhou, Guangdong, China; 5 Department of Anesthesia Surgery, Liwan Central Hospital, Guangzhou, Guangdong, China; 6 School of Biomedical and Pharmaceutical Sciences, Guangdong University of Technology, Guangzhou, Guangdong, China

**Keywords:** atypical Parkinson’s syndrome, imaging omics, multidimensional diagnostic model, Parkinson’s disease, plasma biomarkers

## Abstract

**Background:**

The early differential diagnosis of Parkinson’s disease (PD) and atypical parkinsonian syndromes (APSs) poses challenges. The current methods, which rely on clinical assessments and single-modal biomarkers, lack sufficient sensitivity and specificity. This study aims to develop a multidimensional model integrating plasma biomarkers, clinical parameters, and neuroimaging radiomic features to improve the accuracy of differentiating PD from APS.

**Methods:**

A total of 150 participants were enrolled in the study, including 56 healthy controls (HC), 54 patients with PD, and 40 patients with APSs. Plasma biomarkers (NFL, GFAP, *α*-syn, and tau), clinical indicators (e.g., disease duration and UPDRS-III scores), and radiomic features (1,316 IBSI-standardized features) from magnetic resonance imaging scans of the midbrain and pons were collected. Core variables were screened using LASSO regression and random forest algorithms, and a multivariate logistic regression model was constructed. The performance of the model was evaluated using receiver operating characteristic curves, calibration curves, and cross-validation.

**Results:**

Plasma GFAP and NFL levels showed a significant gradient change: APS group (GFAP: 89.9 pg./mL; NFL: 77.3 pg./mL) > PD group (GFAP: 47.1 pg./mL; NFL: 50.0 pg./mL) > HC group (GFAP: 22.1 pg./mL; NFL: 37.5 pg./mL; *p* < 0.05). The levels of *α*-syn and tau in the PD and APS groups were significantly higher than those in the HC group (*p* < 0.001), but there was no difference between the two groups. Four core variables (NFL, GFAP, disease duration, and pontine voxel volume) were selected. The area under the curve (AUC) of the combined model for identifying PD and APS was 0.874 (95% confidence interval: 0.801–0.946), which was significantly higher than that of single variables (such as NFL alone AUC = 0.653). Cross-validation confirmed the stability of the model (AUC = 0.843).

**Conclusion:**

This was the first study to integrate plasma NFL/GFAP, clinical disease duration, and pontine radiomic features to construct a high-precision PD-APS differential model (AUC > 0.87), addressing the limitations of traditional single-mode approaches. The gradient changes in GFAP and the APS-specificity of NFL serve as key biomarkers. Thus, this multidimensional framework provides a practical diagnostic tool for resource-limited scenarios.

## Introduction

The differential diagnosis of Parkinson’s disease (PD) and atypical Parkinsonian syndromes (APSs) is a major challenge in clinical practice. Both are characterized by core symptoms, such as bradykinesia and myotonia; however, their pathological mechanism and disease progression are significantly different. PD is marked by the degeneration of dopaminergic neurons in the substantia nigra and the aggregation of *α*-synuclein (*α*-syn) (Jankovic and Tan, 2020), whereas APSs (such as multiple system atrophy, progressive supranuclear palsy, and cortical basal ganglia degeneration) are involved in tauopathy and glial cell lesions (Boxer et al., 2017; Komori, 1999). APSs also respond poorly to dopaminergic treatment and have a worse prognosis (Kou et al., 2025a). Due to the overlap of clinical features, the differential diagnosis of PD and APSs is challenging, resulting in a misdiagnosis rate of 24–50% in clinical practice (Tönges et al., 2022). However, unlike PD, APSs show faster progression and a functional impairment prognosis, requiring different treatments. Therefore, accurately distinguishing between PD and APSs is essential for optimizing treatment and patient management.

Currently, PD and APS diagnoses mainly rely on clinical criteria (such as those from the Movement Disorder Society (MDS)), which integrate motor assessments, autonomic features, and neuroimaging. However, these approaches face limitations in sensitivity and specificity, especially during prodromal phases where symptoms mimic idiopathic PD. Advances in biomarker research have shifted the focus toward fluid and imaging modalities to enhance diagnostic precision. Differences in metabolic pattern neuroimaging techniques like ^18^F-FDG PET can assist in differentiating PD from APSs (Nerattini et al., 2025; Zhao et al., 2024; Schröter et al., 2022); however subtype characteristics overlap when these methods are used alone, and they are costly. Among fluid biomarkers, cerebrospinal fluid (CSF) indicators, such as neurofilament light chain (NFL), glial fibrillary acidic protein (GFAP), and tau protein, have been proven effective (Xiang et al., 2022; Kou et al., 2025b); however, their acquisition requires invasive lumbar puncture, which limits their clinical application and popularization. Notably, the increase in the use of blood biomarkers for identifying diseases and disorders provides a new direction for non-invasive diagnosis. Plasma NFL levels, a universal marker of axonal injury, are significantly higher in APS patients (especially those with MSA and PSP) than in PD patients, and is positively correlated with disease severity (Kou et al., 2025b). Plasma GFAP reflects astrocyte activation and shows specific elevations in MSA (Katzdobler et al., 2024). Although *α*-syn is closely related to the core pathology of PD, it may also be abnormally elevated or aggregated in non-PD diseases, such as MSA, which may limit its specificity as a single biomarker (Bougea et al., 2025). Therefore, future diagnostic strategies need to combine comprehensive analyses of clinical assessments, imaging features, and multimodal fluid biomarkers (such as NFL, GFAP, and *α*-syn) to overcome the shortcomings of existing methods and improve the accuracy of differentiation.

Presently, the differential diagnosis of PD and APSs mostly focuses on a single blood index or imaging index, and relatively few studies have been conducted on multi-omics combinations. Due to the limitations of single markers, researchers have begun to explore multi-dimensional integration strategies. For example, the combination of 18F-FDG positron emission tomography (PET) and a deep-learning radiomics model is used to identify PD and APSs. The area under the curve (AUC) of PET is more than 0.9 (Zhao et al., 2024), and the metabolic characteristics of brain regions can be extracted and complemented with blood markers, such as NFL and GFAP. The purpose of this study was to construct a comprehensive multi-dimensional diagnostic model, which integrates plasma NFL, GFAP, tau, *α*-syn levels, combined with clinical parameters, such as Hoehn-Yahr stage and UPDRS scores, as well as neuroimaging features, to achieve an accurate identification of PD and APSs. We hypothesize that integrating these three modalities—plasma biomarkers (NFL, GFAP, tau, *α*-syn), clinical parameters, and midbrain/pons radiomic features—will capture the distinct pathophysiological signatures of PD and APSs (e.g., axonal injury, astrocyte activation, and structural changes in key brain regions) more comprehensively than single-modal approaches, thereby significantly improving the sensitivity and specificity of differential diagnosis. This method can not only overcome the limitations of a single modality, but also capture the heterogeneity of diseases, helping clinicians to grasp the key diagnostic features, and promoting the transformation of clinical applications.

## Methods

### Participants

This cross-sectional study enrolled 150 participants, including 54 with PD, 40 with APSs, and 56 age- and sex-matched healthy controls (HCs) in the Department of Neurology (inpatient and outpatient clinics), Jinan University Affiliated Guangdong Second Provincial General Hospital. PD patients met the MDS Clinical Diagnostic Criteria for PD (2015) (Postuma et al., 2015). MSA was diagnosed according to the MDS Diagnostic Criteria for MSA (2022) (Wenning et al., 2022), and a PSP diagnosis was established according to the International Parkinson and Movement Disorder Society Clinical Criteria for PSP (2017) (Höglinger et al., 2017). CBD was diagnosed according to the Diagnostic Criteria for Corticobasal Degeneration proposed by Armstrong et al. (2013). The APS group consisted of the following subtypes: 25 patients with MSA, 10 with PSP, and 5 with CBD. HCs were asymptomatic volunteers undergoing routine health examinations at the same hospital, and matched for age, sex, and education level. All HCs were screened to exclude neurological and psychiatric disorders, major systemic diseases (e.g., cerebrovascular disease, dementia, and malignancy), and had normal cognitive assessments and brain imaging (magnetic resonance imaging (MRI) or computed tomography). All of the participants underwent serum biomarker testing, clinical assessments (e.g., MDS-Unified Parkinson’s Disease Rating Scale Part III), and structural MRIs. The study protocol was approved by the Ethics Committee of Guangdong Second Provincial General Hospital. Written informed consent for scientific research was obtained from all of the subjects prior to enrollment.

### Clinical assessments

Demographic characteristics (sex, age, and education level) and disease duration were collected for all of the patients with APSs and PD. Global cognitive function was assessed using the Mini-Mental State Examination (MMSE). Disease staging for PD and PSP patients was documented using the modified Hoehn-Yahr (H-Y) stage (Hoehn and Yahr, 2001), while motor symptom severity in PD patients was quantified using the MDS-Unified Parkinson’s Disease Rating Scale Part III (MDS-UPDRS-III) (Goetz et al., 2008), with evaluations performed ≥ 12 h after the last dopaminergic medication. Anxiety symptoms, depressive states, and sleep disturbances were screened using the Hamilton Anxiety Rating Scale (HAMA), Hamilton Depression Rating Scale (HAMD), and Pittsburgh Sleep Quality Index (PSQI), respectively. The intensity of dopaminergic therapy was standardized by recording levodopa equivalent daily doses (LEDDs).

### Blood collection and measurement of NFL, GFAP, tau, and *α*-syn levels

Fasting venous blood was collected in an EDTA-anticoagulated tube (purple cap) and centrifuged at 3500 × g at room temperature for 5 min within 2 h. The plasma was separated and sub-packed at 500 μL per tube and frozen at ˗80 °C. Plasma NFL (Batch No.: L212AD0356 / L423AD0366), *α*-syn (Batch No.: L212AD0354 / L423AD0363), GFAP (Batch No.: L212AD0355 / L423AD0365), and total tau (Batch No.: L212AD0353 / L423AD0364) levels were measured using ELISAs. All of the samples were tested in multiple wells. The intra-assay coefficient of variation was less than 10%, and the absorbance was read at 450 nm. The experimental personnel were blinded to the diagnostic information of the subjects.

### MRI data acquisition

Brain MRI scans were acquired using a Philips Ingenia 3.0 T full-digital MRI scanner with standardized protocols for T1-weighted (TR/TE = 2300/2.98 ms, 1 mm slice thickness, no gap), T2-weighted (TR/TE = 9000/94 ms, 1 mm slice thickness, no gap), and T2-fluid-attenuated inversion recovery (FLAIR) (TR/TE = 9000/94 ms, 1 mm slice thickness, no gap) sequences. Two neuroradiologists with ≥ 8 years of clinical experience manually delineated the midbrain from the inferior colliculi to the cerebral peduncles and pons from the pontomedullary junction to the midbrain-pontine junction on axial T2-weighted images using 3D Slicer software (version 5.2.2), with inter-rater consistency ensured through independent delineation followed by consensus review. Radiomic features were extracted from each region of interest (ROI) using PyRadiomics (version 3.0.1), yielding a total of 1,316 International Biomedical Standardization Initiative (IBSI)-compliant features, including first-order statistics, shape-based features, texture features [gray-level co-occurrence matrix (GLCM), gray-level size-zone matrix (GLSZM)], and wavelet-transformed features. All of the extractions strictly adhered to IBSI guidelines to ensure reproducibility and interoperability across the datasets.

### Statistical analysis

All of the statistical analyses were performed using R software (version 4.3.2) with packages including tidyverse,^1^ glmnet,^2^ randomForest,^3^ and pROC.^4^ Continuous variables are presented as mean ± standard deviation (SD) or median [interquartile range] based on normality, while categorical variables are reported as counts (percentages). Group comparisons were conducted using *t*-tests for normally distributed data or Mann–Whitney U tests for non-normally distributed data. Chi-squared tests were used for categorical variables. Multiple comparisons were adjusted using the Benjamini–Hochberg method. Plasma biomarker distributions were assessed for normality using the Shapiro–Wilk test and visual inspections (e.g., Q-Q plots) (Supplementary Table S1 and Supplementary Figure S1). As these biomarkers exhibited non-normal distributions, they were presented as medians [IQR] in descriptive statistics. However, raw values were used in subsequent statistical analyses, including logistic regression models, to preserve clinical interpretability of odds ratios and align with common practices in biomarker studies where transformations may complicate threshold determinations.

For PD and APS subgroups, variables associated with the outcome were first identified using univariate logistic regression, followed by feature selection using LASSO regression and random forest algorithms. Variables selected by both methods were included in the final model. Variance inflation factors (VIFs < 5) were used to assess multicollinearity, and restricted cubic splines (RCSs) were applied to model nonlinear relationships. A multivariate logistic regression model was constructed, and its performance was evaluated using receiver operating characteristic (ROC) curves. The area under the curve (AUC) and Youden’s index were calculated to determine the optimal threshold.

The diagnostic value of NFL in distinguishing the combined disease group (PD + APS) from HCs was analyzed using the same approach, reporting AUC, sensitivity, specificity, and the optimal cut-off value. Statistical significance was set at *p* < 0.05. All of figures were generated using the ggplot2 package.

## Results

### Demographic and clinical characteristics

A total of 150 subjects were included in this study, including HCs (N = 56), PD patients (N = 54) and APS patients (N = 40, including multiple system atrophy, progressive supranuclear paralysis, and other subtypes). The analysis of demographic characteristics showed no significant differences in age (*p* = 0.320), sex composition (*p* = 0.956), or years of education (*p* = 0.091) among the three groups. Regarding the clinical characteristics, the duration of disease in PD patients (median 5.00 years, interquartile range (IQR) 2.00–8.00 years) was significantly longer than that in APS patients (2.00 years, IQR 1.00–4.25 years, *p* < 0.001). Significant differences were also found between the PD and APS groups in LEDDs (*p* = 0.005), Hoehn and Yahr stage (*p* = 0.043), UPDRS III scores (*p* = 0.007), and HAMA scores (*p* = 0.046). In contrast, simple mental state examination scores (MMSE, *p* = 0.220) and PSQI scores (*p* = 0.104) were not statistically different among the three groups (HCs had no disease course or treatment-related indicators, and only the patient group was compared during the analysis). The detailed demographic, clinical information, and plasma biomarker levels in each group are shown in Table 1.

**Table 1:** The demographic characteristics, clinical information, and plasma biomarker levels of each group.

Variable	HC (*N* = 56)	PD (*N* = 54)	APS (*N* = 40)	*p*-value
Demographics				
Age, years	66.0 [60.8, 71.3]	67.5 [55.3, 74.0]	70.0 [60.0, 77.3]	0.320
Gender(Female)	29 (52%)	28 (52%)	22 (55%)	0.956
Education, years	9.00 [6.00, 12.0]	6.00 [6.00, 9.00]	6.00 [6.00, 13.0]	0.091
Plasma Biomarkers (pg/mL)				
NFL	37.5 [22.7, 78.1]	50.0 [25.0, 88.4]	77.3 [30.1, 121]	0.009*
Tau	19.8 [7.07, 49.5]	88.3 [40.0, 172]	101 [59.2, 160]	<0.001*
GFAP	22.1 [5.85, 41.4]	47.1 [23.2, 86.3]	89.9 [36.9, 345]	<0.001*
*α*-Synuclein	42.5 [24.1, 62.4]	288 [170, 508]	372 [249, 527]	<0.001*
Clinical characteristics				
Disease Duration, years	NA	5.00 [2.00, 8.00]	2.00 [1.00, 4.25]	<0.001*
LEDD, mg/day	NA	560 [375, 664]	325 [169, 556]	0.005*
Hoehn & Yahr Stage	NA	3.00 [2.00, 3.00]	3.00 [2.38, 4.00]	0.043*
UPDRS III	NA	32.5 [24.3, 37.0]	38.5 [29.8, 47.0]	0.007*
MMSE	26.0 [24.0, 27.0]	24.0 [21.0, 27.8]	24.0 [19.0, 28.0]	0.220
PSQI	10.0 [7.75, 12.0]	10.0 [4.50, 13.0]	10.0 [5.00, 12.0]	0.104
HAMA	7.00 [6.00, 9.00]	10.5 [5.00, 13.0]	10.0 [4.75, 12.3]	0.046*
HAMD	8.00 [7.00, 12.0]	9.00 [6.25, 13.0]	7.00 [5.00, 11.3]	0.140

NFL, Neurofilament Light Chain; GFAP, Glial Fibrillary Acidic Protein; Tau, Microtubule-associated protein tau; LEDD, Levodopa Equivalent Daily Dose; MMSE, Mini-Mental State Examination; UPDRS III, Unified Parkinson’s Disease Rating Scale Part III; PSQI, Pittsburgh Sleep Quality Index; HAMA, Hamilton Anxiety Rating Scale; HAMD, Hamilton Depression Rating Scale. *p < 0 0.05; *p*-value is the result of Kruskal–Wallis test.

### Plasma marker levels in different diagnostic groups

**Figure 1:**
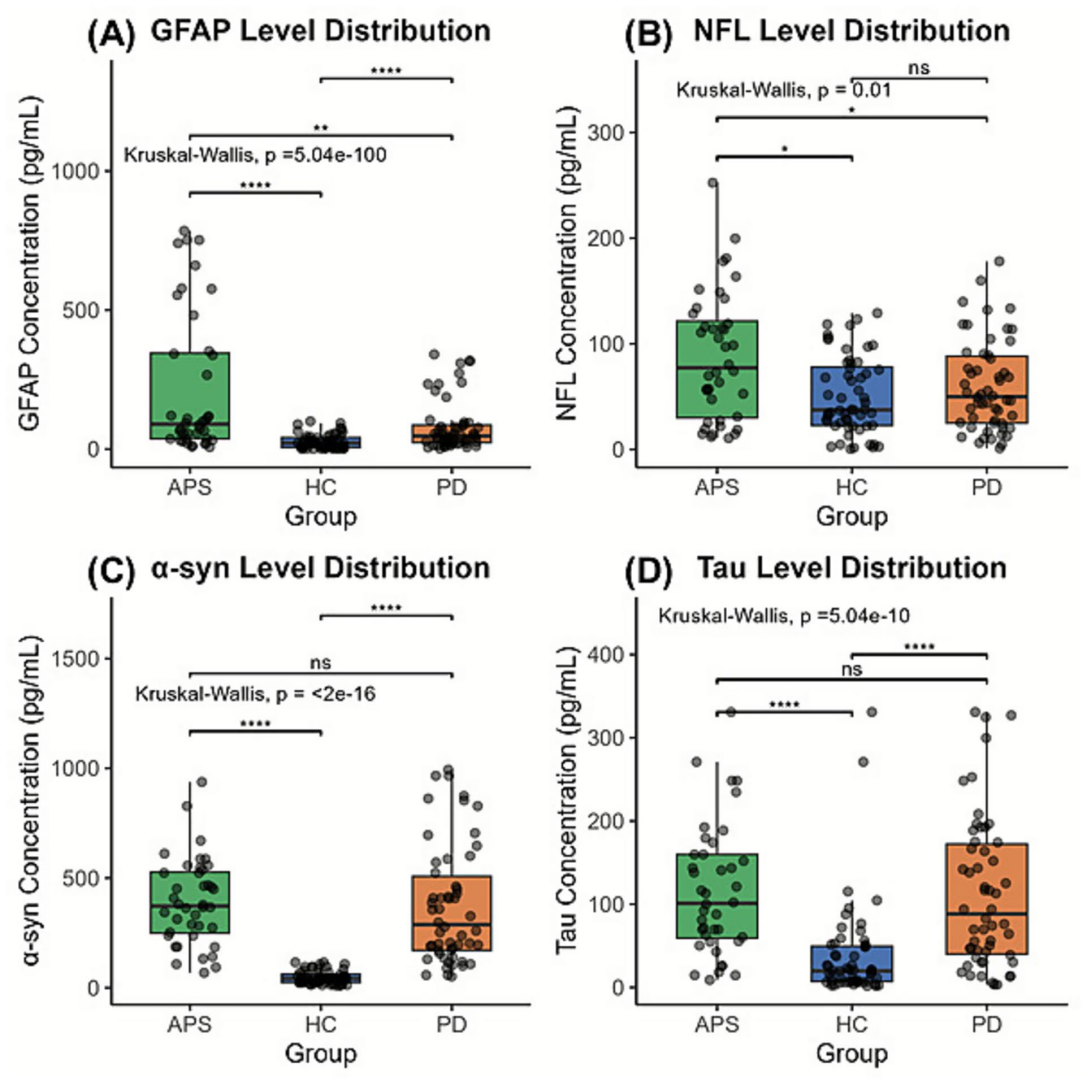
**(A–D)** Comparisons of plasma biomarker levels. Significance: ****p < 0.001, **p < 0.01, *p < 0.05. HC, healthy control; PD, Parkinson’s disease; APS, atypical Parkinsonian syndromes; NFL, neurofilament light; GFAP, glial fibrillary acidic protein; *α*-syn, *α*-synuclein; Tau, microtubule-associated protein tau.

The plasma marker levels in different diagnostic groups were analyzed after adjusting for age, sex, and disease duration, (Figure 1). The analysis of plasma biomarkers showed significant differences in the distribution of GFAP, NFL, *α*-syn, and tau protein among groups (Kruskal–Wallis test, all *p* < 0.05).

GFAP levels were significantly different among the three groups (*p* = 3.17e-09). Pairwise comparison showed a median GFAP level of 89.9 pg./mL in the APS group, which was significantly higher than that in the PD group (47.1 pg./mL, *p* < 0.001) and HC group (22.1 pg./mL, *p* < 0.001). GFAP levels in the PD group were also significantly higher than in the HC group (*p* < 0.01), showing a gradient trend of HC < PD < APS.

The overall NFL levels were significantly different (*p* = 0.00952), and the pairwise comparison highlighted the specificity of the APS group. The median NFL level in the APS group was 77.3 pg./mL, which was significantly higher than in the PD group (50.0 pg./mL, *p* < 0.05) and in the HC group (37.5 pg./mL, p < 0.05); however, there was no significant difference between the PD group and the HC group (*p* > 0.05), suggesting that the increase in NFL was a characteristic change of APS.

The *α*-syn levels were significantly higher in the PD group (288 pg./mL) and the APS group (372 pg./mL) than that in the HC group (42.5 pg./mL, *p* > 0.05), indicating that *α*-syn elevation was a common pathological feature of PD and APS, with similar increases.

Microtubule-associated tau protein levels were also very significantly different between the groups (*p* = 5.04e-10). Pairwise comparison showed that the consistency of PD and APS increased. The median tau level in the HC group was the lowest (19.8 pg./mL), and those in the PD group (88.3 pg./mL) and the APS group (101 pg./mL) were significantly higher than in the HC group (*p* < 0.001). However, there was no significant difference between the PD group and the APS group (*p* > 0.05), suggesting that the two have common tau protein characteristic abnormalities, and both levels were significantly different from those in HCs.

### Screening analysis of core indicators between PD and APS

#### Univariate logistic regression analysis

The univariate logistic regression results showed that multiple indicators were statistically associated with PD and APS (Table 2). Among the blood biomarkers, elevated NFL (*p* = 0.015) and GFAP levels (*p* = 0.003) were significantly correlated with each group [odds ratio (OR) = 1.011, 1.004, 95% confidence interval CI: > 1]. Among the clinical characteristics, shorter disease duration (OR = 0.754, *p* = 0.001), lower LEDD (OR = 0.998, *p* = 0.011), and higher UPDRS III scores (OR = 1.063, *p* = 0.006) were significantly associated with each group. In terms of imaging features, significant differences in midbrain voxel volume (OR = 0.999, *p* = 0.004), the run-length inhomogeneity of pontine T2-FLAIR sequence (OR = 0.999, *p* = 0.007), midbrain T1 weighted image kurtosis (OR = 0.850, *p* = 0.017), and the 90th percentile of pontine T2 signal intensity were found (OR = 1.002, *p* = 0.015). Demographic characteristics (age, gender, and years of education), tau protein, *α*-syn, and other indicators showed no statistical correlations (all *p* > 0.05).

**Table 2:** Results of univariate logistic regression analysis between PD and APS groups.

Variable	SE	*z*	*β*	OR (95% CI)	*p*-value
Demographics					
Age	0.019	1.365	0.026	1.027 (0.989–1.068)	0.172
Gender (male)	0.419	−0.302	−0.127	0.881 (0.386–2.002)	0.762
Education	0.043	0.669	0.029	1.029 (0.946–1.121)	0.503
Blood biomarkers					
NFL	0.004	2.444	0.011	1.011 (1.002–1.020)	0.015*
Tau	0.002	0.217	0.001	1.001 (0.996–1.005)	0.828
GFAP	0.001	2.936	0.004	1.004 (1.002–1.008)	0.003*
*α*-Synuclein	0.001	0.409	0.000	1.000 (0.999–1.002)	0.683
Clinical characteristics					
Disease duration	0.087	−3.243	−0.283	0.754 (0.622–0.876)	0.001*
LEDD	0.001	−2.543	−0.002	0.998 (0.996–0.999)	0.011*
MMSE	0.025	−0.719	−0.018	0.982 (0.933–1.032)	0.472
Hoehn & Yahr Stage	0.199	1.838	0.367	1.443 (0.985–2.167)	0.066
UPDRS III	0.022	2.732	0.061	1.063 (1.019–1.112)	0.006*
PSQI	0.042	−0.937	−0.039	0.962 (0.885–1.043)	0.349
HAMA	0.033	−1.054	−0.035	0.966 (0.902–1.029)	0.292
HAMD	0.033	−1.691	−0.056	0.946 (0.882–1.004)	0.091
Image feature					
VoxelVolume_midbrain	0.001	−2.858	−0.001	0.999 (0.999–1.000)	0.004*
T2_Flair_RunLengthNonUniformity_pons	0.001	−2.707	−0.001	0.999 (0.999–1.000)	0.007*
VoxelVolume_pons	0.001	−2.688	−0.001	1.000 (0.999–1.000)	0.007*
SurfaceArea_midbrain	0.001	−2.559	−0.002	0.998 (0.996–0.999)	0.011*
T2_90Percentile_pons	0.001	2.423	0.002	1.002 (1.001–1.005)	0.015*
T1_Kurtosis_midbrain	0.068	−2.388	−0.162	0.850 (0.737–0.965)	0.017*

VoxelVolume_midbrain, Midbrain voxel volume; T2_Flair_RunLengthNonUniformity_pons, The run-length inhomogeneity of pontine T2-FLAIR sequence; VoxelVolume_pons, Pontine voxel volume; SurfaceArea_midbrain, Midbrain surface area; T2_90Percentile_pons, 90th percentile of pontine T2 signal intensity; T1_Kurtosis_midbrain, Midbrain T1 weighted image kurtosis; SE, Standard Error; *z*, *z*-statistic/*z*-score; *β*, Beta coefficient.

*p < 0.05, *p*-value is the result of *z*-test. Beta coefficient.

#### LASSO regression variable screening

**Figure 2:**
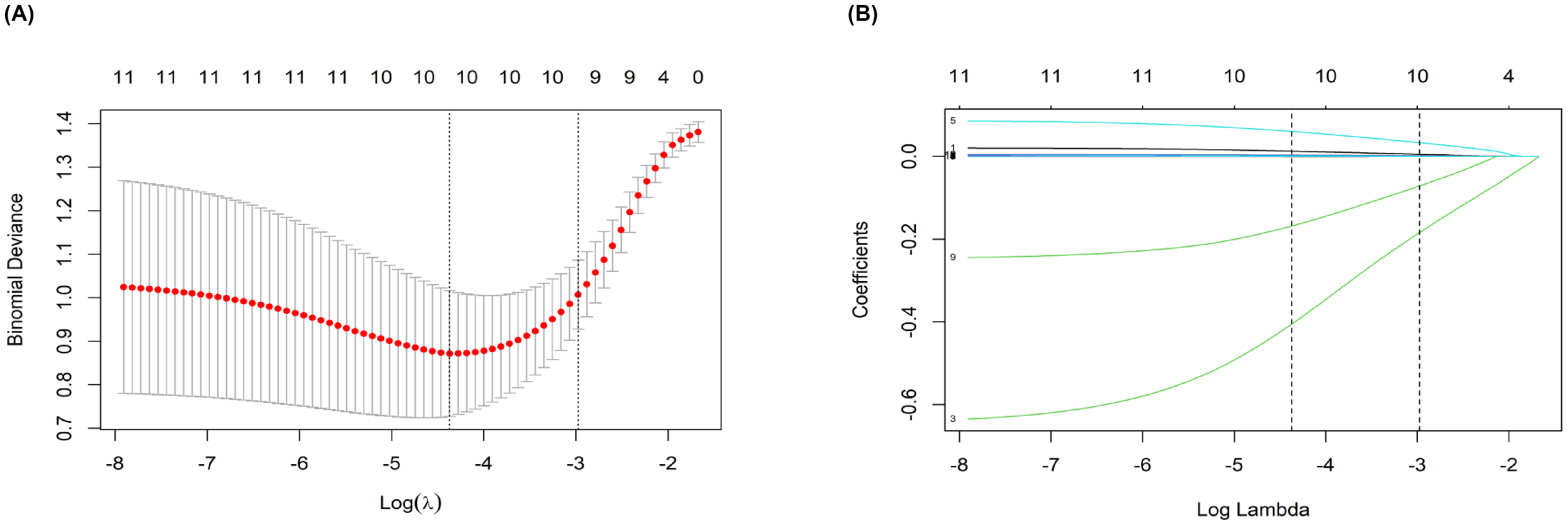
**(A)** The longitudinal axis is a binomial deviation, and the transverse axis is Log (*λ*). The optimal λ (Log (λ) = −4.5) was screened by 1-SE rule (dotted line), and the model fitting and generalization were balanced. **(B)** The vertical axis is the normalized coefficient, and the horizontal axis is Log (λ). The curve shows the variable contraction trajectory, and 9 core variables are retained under the optimal λ (dotted line).

Eighteen candidate variables in univariate logistic regression were included in the LASSO regression to reduce multicollinearity and focus on key identification indicators. The optimal regularization parameters were determined using the deviation-*λ* curve (Figure 2A). The coefficient path diagram (Figure 2B) visually presents the variable shrinkage process, compresses the variables, and finally retains nine core variables: NFL, GFAP, duration of disease, UPDRS III scores, midbrain voxel volume, pontine voxel volume, midbrain T1-weighted image kurtosis, 90th percentile of pontine T2 signal intensity, and the run-length inhomogeneity of the pontine T2-FLAIR sequence.

#### Random forest variable screening

**Figure 3:**
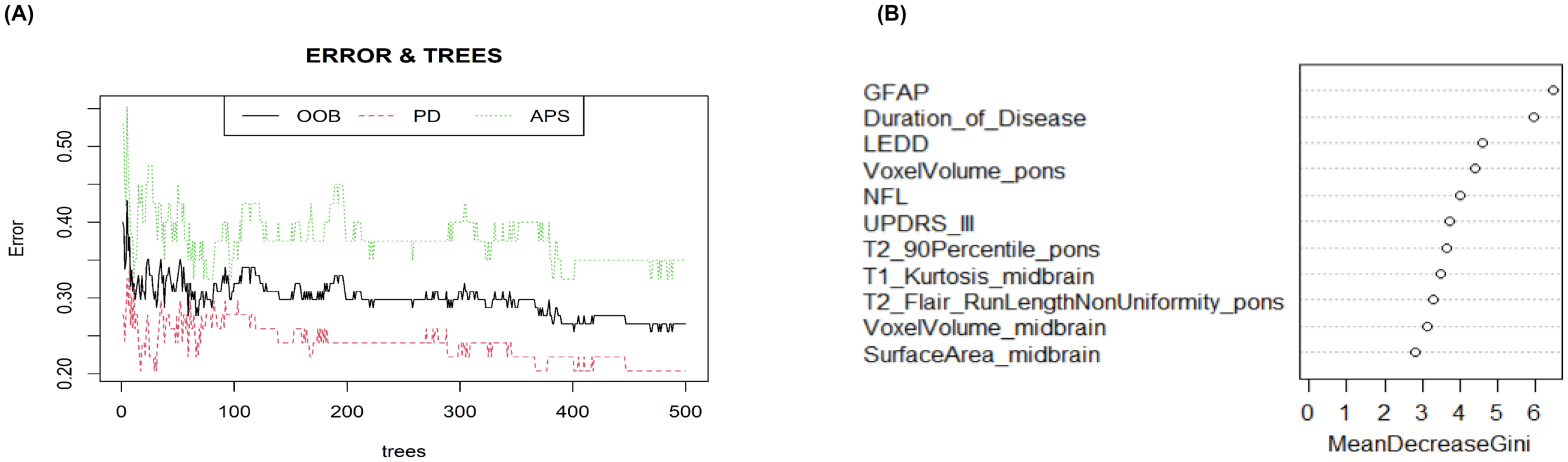
**(A)** The horizontal axis is the number of decision trees, and the vertical axis is the classification error. The errors of OOB, PD, and APS converge with the increase of tree, and the model is stable when the tree is 500. **(B)** Screening the top 5 core variables with the MeanDecreaseGini indicator on the right graph.

Similarly, 18 candidate variables in univariate logistic regression were used to construct a random forest model. The error-tree convergence curve (Figure 3A) showed that when the number of decision trees was ≥ 300, the classification errors of out-of-bag and PD and APS tended to be stable. Therefore, a 500-tree optimization model was set. The importance of variables was evaluated by MeanDecreaseGini (Figure 3B), and the top five variables were selected from high to low index values: GFAP (6.453), disease duration (5.930), LEDD (4.613), pontine voxel volume (4.379), and NFL (3.985). The results showed that the MeanDecreaseGini values of GFAP and disease duration were the highest, suggesting that they contributed the most to identifying the two groups.

#### Verification of core variables and analysis of correlation characteristics

The intersection analysis of the LASSO regression and random forest screening results showed that four variables constituted the core indicators for identifying PD and APS: NFL, GFAP, disease duration, and pontine voxel volume.

The collinearity test of the core variable set showed that the VIF of all of the variables was less than 5, suggesting no significant multicollinearity, which could be used for subsequent model construction. Further analysis of the correlation between variables and outcomes showed that disease duration and pontine voxel volume were linearly correlated with each group, while NFL and GFAP showed non-linear correlation characteristics, suggesting that these two biomarkers may be involved in the pathological identification of PD and APS through more complex dose effects.

#### Joint model construction and performance verification

Based on the four core variables (NFL, GFAP, disease duration, and pontine voxel volume) screened by LASSO and random forest, the nonlinear correlation between NFL and outcome was analyzed using RCS, and an RCS-logistic regression model was constructed to verify performance from the discrimination, calibration, and clinical benefit perspectives.

#### Nonlinear dose-effect relationship of NFL

**Figure 4:**
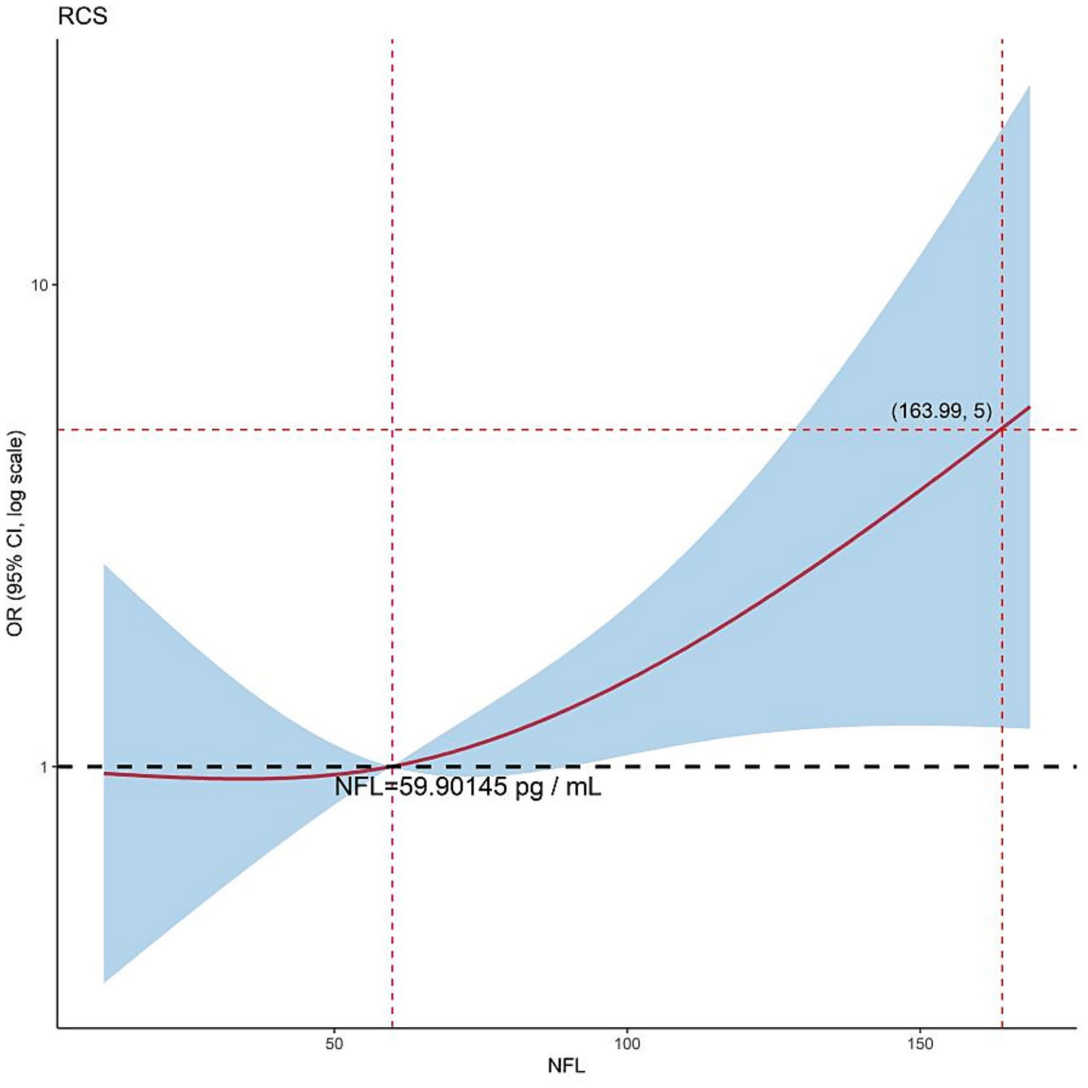
Restricted cubic spline curves for the discriminative ability of NFL to distinguish PD from APS.

The RCS curve of NFL levels (OR on the logarithmic scale) revealed the nonlinear correlation characteristics of its identification of PD and APS. When NFL levels were lower than 59.90145 pg./mL (the cut-off value of the vertical dotted line marker), the OR was close to 1 (95% CI included 1), suggesting that low NFL concentrations did not significantly contribute to identifying the two groups. When the NFL value exceeded the cut-off value, the OR value increased exponentially with increases in concentration (the slope of the curve increased sharply), with an OR of > 5 at high NFL concentrations (such as > 163.99 pg./mL), indicating that the efficiency of identifying APS increased sharply with increases in concentration (Figure 4).

#### Model discrimination ability: ROC curve comparison

**Figure 5:**
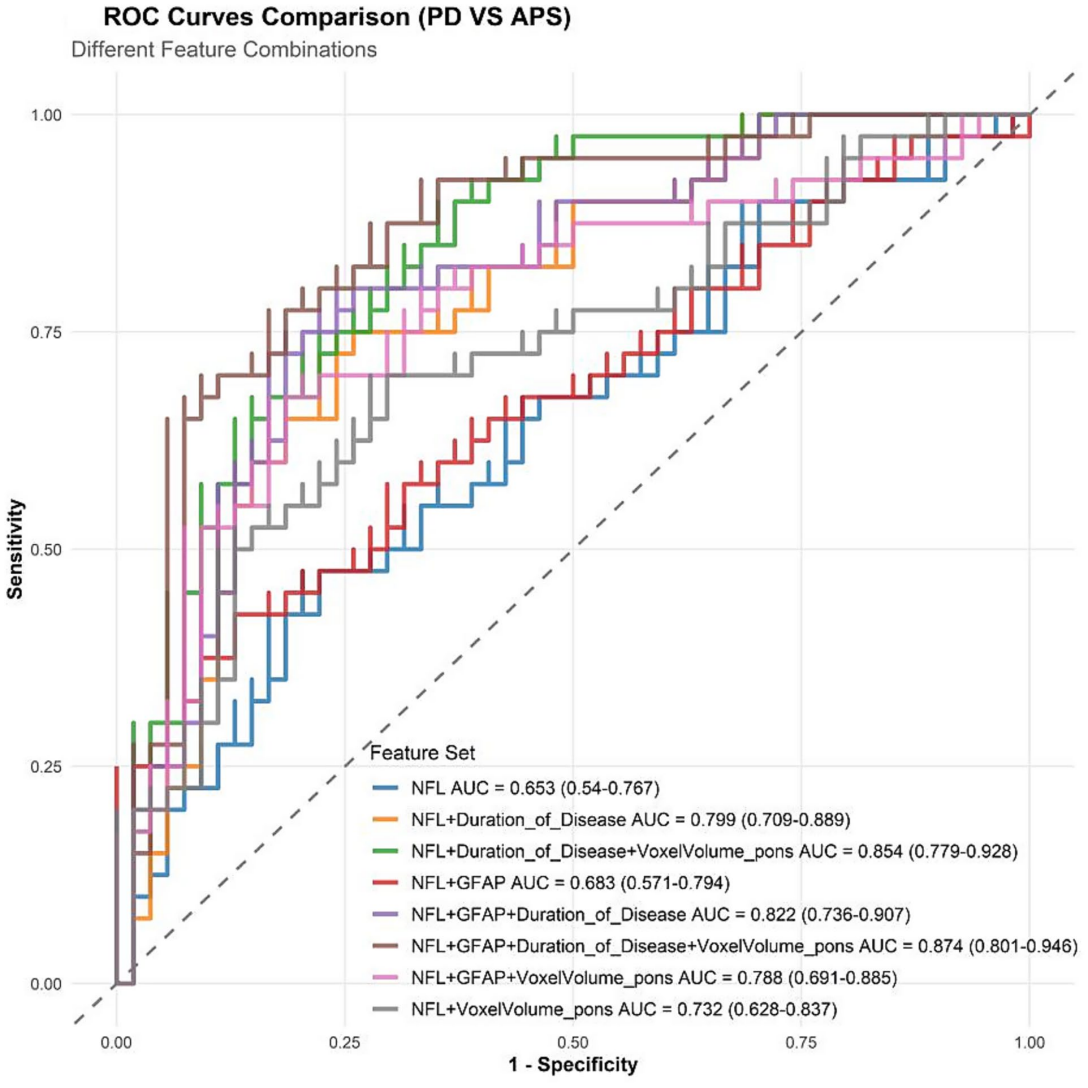
Comparison of ROC curves of different feature combinations in the identification of PD and APS.

ROC analysis of different feature combinations showed that the four-variable combined model (NFL + GFAP + disease duration + pontine voxel volume) had the best identification efficiency, with an AUC of 0.874 (95% CI: 0.801–0.946), which was significantly higher than that of the univariate (NFL alone, AUC = 0.653), bivariate (such as NFL + disease duration, AUC = 0.799), and three-variable combination models (such as NFL + GFAP + disease duration, AUC = 0.822). The ability of the combined model to distinguish PD from APS was significantly improved compared to the other models (Figure 5).

#### Model calibration capability: calibration curve and cross validation

**Figure 6:**
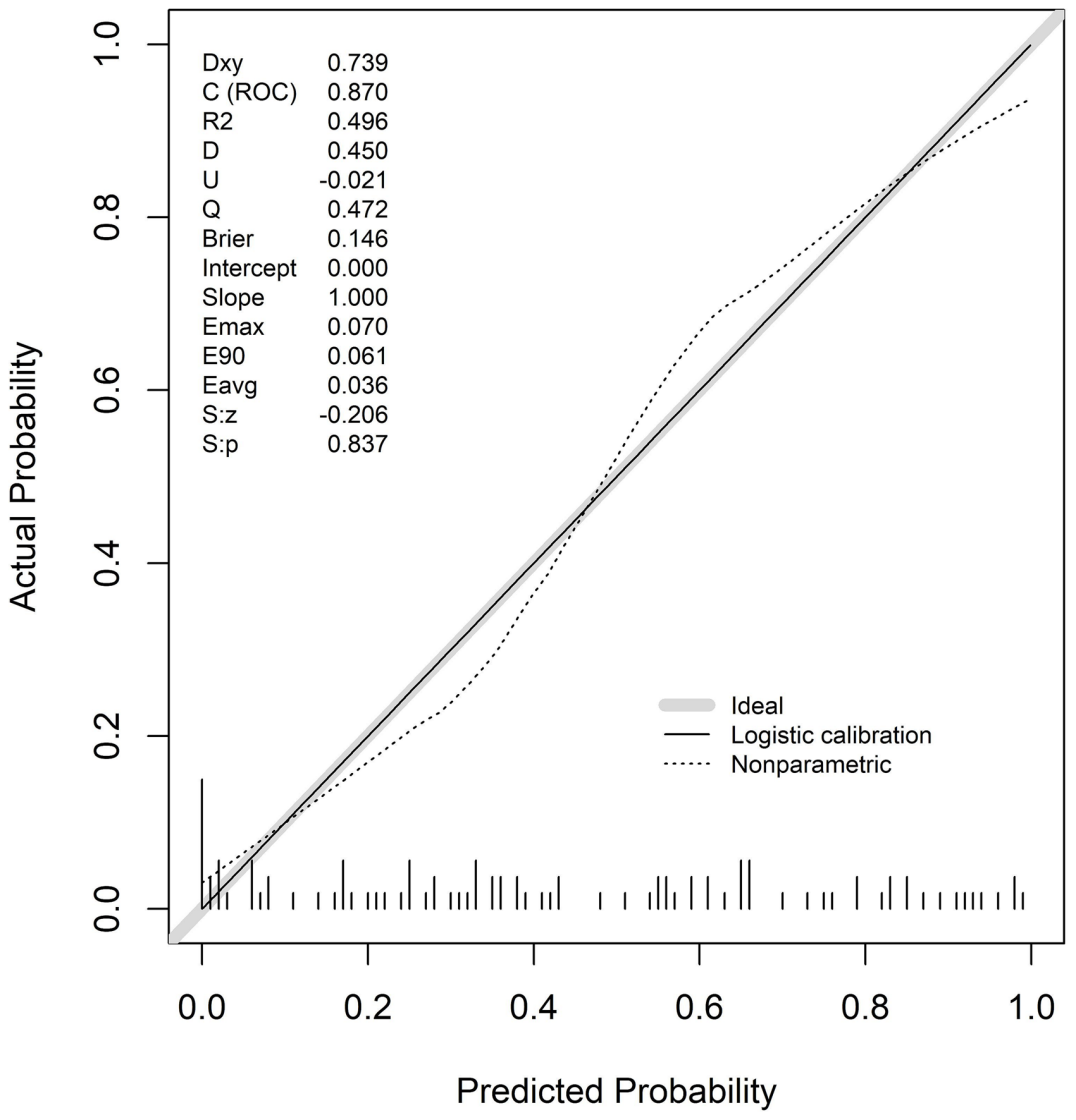
Calibration curve and performance index of RCS-logistic.

Calibration curve (Figure 6): The predicted probability of the model fit well with the actual prevalence probability. The C-index (0.870) value was close to the ideal value. The Brier score (0.146) indicated that the prediction error was small. The logistic and non-parametric calibration curves were close to the diagonal, and with no obvious calibration deviation.

Cross-validation (Table 3): In 400 5-fold cross-validations, the changes in AUC (0.843), R2 (0.244) and discrimination index values (D = 0.222) after calibration were less than 0.3, indicating that the model was generalized and stable across different data subsets.

**Table 3:** Seven indicators combined with 400 times 5-fold cross-validation for in-depth evaluation of the RCS-logistic model.

Indicator	Modeling data value	Calibrated value	Δ
AUC/C-index	0.86967593	0.84303969	−0.02663623
R^2^	0.49626443	0.24394511	−0.25231932
Discrimination_Index(D)	0.45045323	0.22247298	−0.22798025
U.test	−0.02127660	0.08407114	0.10534774
Brier.Score	0.14644159	0.16805369	0.02161209
Maximum.Deviation	0.06989871	0.30918684	0.23928813
Minimum.Deviation	0.03599203	0.13723395	0.10124192

## Discussion

The purpose of this study was to establish a multimodal diagnostic framework to solve the clinical problem of the differential diagnosis of PD and APS by integrating clinical evaluation, plasma biomarkers, and radiomics features from midbrain and pons MRI scans. The main findings showed that although demographic variables, such as age, gender, and education level, did not differ between the groups, clinical indicators, such as disease duration, LEDD, H-Y stage, MDS-UPDRS-III scores, and HAMA scores, could distinguish PD from APS, while tau protein and *α*-syn plasma biomarkers were not independently associated. In particular, logistic regression analysis showed that the disease duration (OR = 0.754) and midbrain T1-weighted image kurtosis (OR = 0.850) had significant ORs, emphasizing their discriminatory ability and highlighting the potential of the framework to improve early diagnostic accuracy.

These results are consistent with the hypothesis that PD and APSs exhibit different clinical, neuroimaging features and biomarker patterns, reflecting potential pathophysiological differences. The longer duration of PD relative to APS may be due to the slower loss of dopaminergic neurons in the substantia nigra, whereas APSs, such as MSA and PSP, show rapid multi-system degeneration, in which tau-mediated cytoskeleton destruction dominates. The high LEDD and H-Y stages in PD indicate a compensatory response to chronic nigrostriatal deficits, whereas APS exhibits more severe motor symptoms at the early stage, with higher UPDRS-III scores despite a shorter disease duration. No differences were seen in the MMSE and PSQI scores among the groups, indicating that cognitive and sleep impairments are secondary or overlapping features that have not yet shown divergence in these cohorts. This finding may be attributed to the mitigation of shared frontal-basal ganglia circuit vulnerabilities through neuroplasticity (Aarsland et al., 2021). In terms of neuroimaging, the positive OR for the 90th percentile of the pontine T2 signal intensity implies aggravated microstructural damage, such as gliosis or axonal loss, in APS, which is consistent with the theoretical model that pontine nuclei are involved in postural instability and autonomic dysfunction (Lee et al., 2000). In terms of biomarkers, the elevated levels of GFAP in APS patients compared with PD patients and HCs support the hypothesis that astrogliosis is more prominent in APS, which may reflect a robust glial activation response to extensive neurodegeneration. This gradient (HC < PD < APS) indicates that reactive astrocytes are involved in the effects of APS subtypes, such as multiple system atrophy or progressive supranuclear palsy, where glial pathology leads to axonal damage and synaptic loss, supported by the theoretical framework of neuroinflammation-driven progression (Guo et al., 2023; Katzdobler et al., 2024). Similarly, the specific increase in NFL in APS highlights axonal degeneration as its marker. The pathology of the substantia nigra striatum is more limited in PD patients compared with the rapid subcortical damage seen in APS, and NFL levels are comparable to those in HCs. No differences were found in NFL concentrations between PD patients and HCs, indicating that axonal injury in PD may be more localized or compensated at these stages. Meanwhile, the shared elevation in *α*-syn in both PD and APS reinforces synucleinopathy as a common basis, likely originating from the extracellular release of damaged neurons. In contrast, the increased tau levels in both conditions indicate tau-mediated cytoskeletal instability that deviates from the healthy state, which could be associated with secondary tau pathology in synuclein-dominant disorders. However, neither of these proteins could distinguish between the syndromes. The limited correlation of plasma tau and *α*-syn may be attributed to their intracellular aggregation rather than free circulation, which restricts their value as standalone discriminatory markers but does not negate their role in pathogenesis.

Our findings align with and extend recent high-impact studies in neuroimaging, humoral biomarkers, and multimodal integration, providing contextual validation while highlighting key differences that underscore our methodological innovations in differentiating PD from APS. In neuroimaging, our use of structural T2-weighted radiomics to detect subtle pontine changes builds on Meindl et al.’s (2025) multimodal approach with machine learning for PD prediction (AUC 0.95), offering greater sensitivity to overlooked features compared to resting-state functional MRI. This aligns with Chougar et al.’s (2021) MRI radiomics for PD-APS differentiation (90% accuracy) but differs in our focus on signal intensity over volume measurements, enhancing detection of heterogeneity in the pontine region. For humoral biomarkers, our detection of elevated plasma *α*-syn, tau, NFL, and GFAP aligns with prior reports of higher levels in PD versus APS, as seen in Lin et al. (2018) and Youssef et al. (2023), though differences in specificity may arise from their emphasis on early-stage cohorts or detection methods. This contextualizes our results within Hall et al.’s (2012) CSF-based differentiation (AUC 0.93 for combined biomarkers), where our non-invasive plasma approach enhances clinical utility and augments NFL’s role as a supplementary marker. Similarly, Hansson et al.’s (2017) affirmation of plasma NFL specificity is extended in our study through GFAP integration, while Parnetti et al.’s (2019) use of extracellular vesicle *α*-syn parallels our findings but is surpassed by our direct plasma gradient without isolation. Jabbari et al.’s (2020) link between NFL and motor severity supports our interpretations, with discrepancies in cognitive correlations likely due to staging variations, highlighting the need for standardized cohorts. In multimodal integration, our fusion of pontine T2 radiomics with clinical biomarkers (e.g., UPDRS) extends Seki et al.’s (2019) AI-based MRI classification for PD subtypes, offering APS-specific interpretable features via OR analysis. This aligns with Chougar et al.’s (2021) microstructural changes but differs in model choice (logistic regression vs. deep learning), potentially due to scale, while enhancing performance through signal intensity focus. Bian et al.’s (2023) meta-analysis on radiomics fusion contextualizes our approach, strengthened by plasma biomarkers for better translation. Barthel et al.’s (2015) PET combinations support the *α*-syn and tau synergy in our results, though our plasma method reduces invasiveness. In contrast, Tosun et al.’s (2024) association of *α*-syn with cognitive decline differs from our MMSE findings, emphasizing the value of longitudinal studies to resolve staging-related discrepancies. Overall, this integrative synthesis with prior literature validates our findings’ reliability, while key differences highlight our contributions: the non-invasive fusion of pontine T2 radiomics with plasma NFL, GFAP, *α*-syn, and tau biomarkers overcomes limitations of invasive (e.g., CSF, vesicle isolation) or unimodal methods, advancing clinical differentiation of PD from APS.

Regarding clinical translation, our multimodal diagnostic framework holds promise for practical implementation in routine neurological practice. Plasma biomarker testing, utilizing standard ELISA kits for markers like NFL, GFAP, *α*-syn, and tau, is highly feasible due to its non-invasive nature—requiring only a simple blood draw—and compatibility with existing laboratory infrastructure in most hospitals. This approach minimizes patient burden compared to invasive CSF sampling and can be performed during outpatient visits, with results potentially available within hours to days depending on lab turnaround. Radiomic features from pontine and midbrain MRI scans are accessible via standard 3 T MRI protocols already common in clinical settings; extraction can be facilitated by open-source or commercial radiomics software (e.g., 3D Slicer), with potential for automation through AI plugins to reduce radiologist workload. Clinicians could integrate the model’s outputs—such as probability scores from logistic regression—into diagnostic workflows by using them as adjunctive tools alongside traditional clinical assessments (e.g., UPDRS scoring and history taking). For instance, in cases of diagnostic uncertainty between PD and APS, high model-predicted probabilities could prompt targeted follow-up (e.g., additional imaging or specialist consultation), ultimately aiding in earlier, more accurate diagnoses and personalized treatment planning, such as adjusting LEDD or initiating APS-specific therapies.

As a single-center cross-sectional study, this research has certain limitations. The sample size was relatively small (*n* = 150), including 54 patients with PD, 40 with APS, and 56 healthy controls. Among the 40 APS patients, the breakdown by subtype was as follows: 25with MSA, 10 with PSP, and 5 with CBD. The depth of the differential analysis of APS subtypes, such as MSA, PSP and CBD, was insufficient. This was mainly due to difficulties in recruitment and the slow sample accumulation caused by the requirement of multidisciplinary consultations (neurology, imaging, and pathology departments) to confirm APS cases. Furthermore, by pooling all APS subtypes into a single category for the primary analysis, we introduced potential heterogeneity, as these syndromes have distinct pathophysiologies, clinical presentations, and biomarker profiles (Stamelou and Hoeglinger, 2013; Coughlin et al., 2021). For instance, multiple system atrophy (MSA) often involves prominent autonomic dysfunction (such as orthostatic hypotension and urinary disturbances) and cerebellar features (including ataxia and dysarthria) (Wenning et al., 2022), while progressive supranuclear palsy (PSP) is characterized by 4R tauopathy with hallmark clinical features such as vertical supranuclear gaze palsy, early postural instability with falls, and axial rigidity (Höglinger et al., 2017; Boxer et al., 2017). This pooling approach risks the model primarily identifying broad “non-PD” features rather than subtle, subtype-specific discriminators, which could dilute the framework’s precision in real-world scenarios where distinguishing between specific APS subtypes (e.g., MSA vs. PSP) is clinically relevant for prognosis, management, and potential targeted therapies (Obelieniene et al., 2018). Such heterogeneity may also confound the interpretation of biomarkers like GFAP (reflecting astrocytic activation) and NFL (indicating neuroaxonal damage), as their elevations could vary across subtypes due to differing degrees of glial activation or axonal damage (Katzdobler et al., 2024). The initial focus of the study design was to verify diagnostic efficacy, without including a longitudinal evaluation module, and it was difficult to supplement follow-up data in the later stage. Therefore, long-term data to explore dynamic changes in biomarkers, such as the annual GFAP growth rate, and their association with disease progression, were lacking. Methodologically, although the use of ELISAs for detecting plasma markers (intra-assay CV < 10%) is reliable, the method’s sensitivity is lower than that of the Simoa technique, and may miss subtle differences in the low-concentration range, such as changes in NFL between early PD and HCs. The study also did not include other potentially relevant markers, such as IL-6 and TREM2 and thus, might have missed opportunities to optimize the model. In terms of imaging analysis, the reliance on the manual delineation of pontine and midbrain volumes, despite passing double-observer consistency verification, still carries the risk of subjective bias. In addition, the failure to include functional imaging data, such as ^18^F-DOPA PET dopamine transporter imaging (Nerattini et al., 2025), limited the model’s ability to capture functional differences. To address the concern regarding APS subtype pooling, we conducted supplementary analyses evaluating the model’s performance in pairwise comparisons (Supplementary Table S2 and Supplementary Figure S2). In these analyses, the multimodal framework achieved a highest AUC of 0.859 for PD vs. MSA, 0.944 for PD vs. PSP, and 0.859 for PD vs. CBD. Additionally, the AUC values for the differential diagnosis among MSA, PSP and CBD all exceeded 0.8, suggesting favorable discriminatory power despite the smaller subgroup sizes. However, these results should be interpreted cautiously due to limited statistical power, and larger subtype-specific cohorts are needed for validation.

Future studies should address these limitations by engaging in multi-center collaborations to expand the sample size, especially increasing the number of patients with various APS subtypes, and explore the inclusion of subtype-specific markers, such as MOG, in MSA research. Designing prospective cohorts (e.g., 24-month follow-ups with assessments every 6 months) is crucial to systematically collect dynamic longitudinal data on plasma markers, clinical indicators, and imaging parameters, thereby constructing models for predicting disease progression and prognosis. Upgrading detection technologies, such as adopting the Simoa platform and expanding the coverage of markers, including IL-6 (Ma et al., 2025) and TREM2 (Huang et al., 2024), can improve the sensitivity and comprehensiveness of the analysis. In imaging analysis, developing deep learning-based automated methods, such as using U-Net models for ROI delineation, will help reduce subjective errors and enhance efficiency and objectivity. Meanwhile, integrating functional imaging data, such as PET, will provide more comprehensive pathological information.

## Conclusion

This study showed that a multi-dimensional model integrating plasma NFL, GFAP, disease duration, and pontine voxel volume could distinguish PD from APS with high accuracy (AUC = 0.874) and stability. The key findings highlight the differential value of GFAP (graded elevations in HCs, PD patients, and APS patients) and NFL (APS-specific elevations) as biomarkers. Alpha-syn and tau were found to be common pathological indicators but lacked subtype specificity. The combined model overcomes the limitations of single-mode assessments and captures the biochemical and structural pathological features of the diseases. These results support the clinical value of multi-dimensional integration in improving the diagnostic accuracy of Parkinson’s syndrome, especially in areas with limited resources and the lack of advanced imaging equipment. In the future, the prognostic value of the model should be verified and its applicability in identifying APS subtypes explored in a larger sample-size multi-center cohort and longitudinal follow-up study.

## Data Availability

The raw data supporting the conclusions of this article will be made available by the authors, without undue reservation.

## Footnotes

1
https://github.com/tidyverse/tidyverse

2
https://github.com/cran/glmnet

3
https://github.com/cran/randomForest

4
https://github.com/xrobin/pROC
