# Supplementary material for: Combining plasma biomarkers, clinical parameters, and neuroimaging features for differential diagnosis of Parkinson’s disease and atypical parkinsonian syndromes: a multidimensional modeling approach
Source: Front Aging Neurosci. 2026 Jan 16;18:1727812. doi: 10.3389/fnagi.2026.1727812 (PMC12855463; doi:10.3389/fnagi.2026.1727812)

Table S1 Shapiro-Wilk Normality Test Results for Plasma Biomarkers

Group	Variable	Sample_Size	Statistic	P_Value
PD	NFL	54	0.9348	0.0057
PD	Tau	54	0.9062	0.0005
PD	GFAP	54	0.7425	<0.001
PD	syn	54	0.8861	0.0001
APS	NFL	40	0.9409	0.0369
APS	Tau	40	0.9418	0.0398
APS	GFAP	40	0.7521	<0.001
APS	syn	40	0.9683	0.0318
HC	NFL	56	0.9323	0.0037
HC	Tau	56	0.5780	<0.001
HC	GFAP	56	0.8716	<0.001
HC	syn	56	0.8955	0.0002

Figure S1 Q-Q Plots for Blood Biomarkers (Non-Normal Distribution)

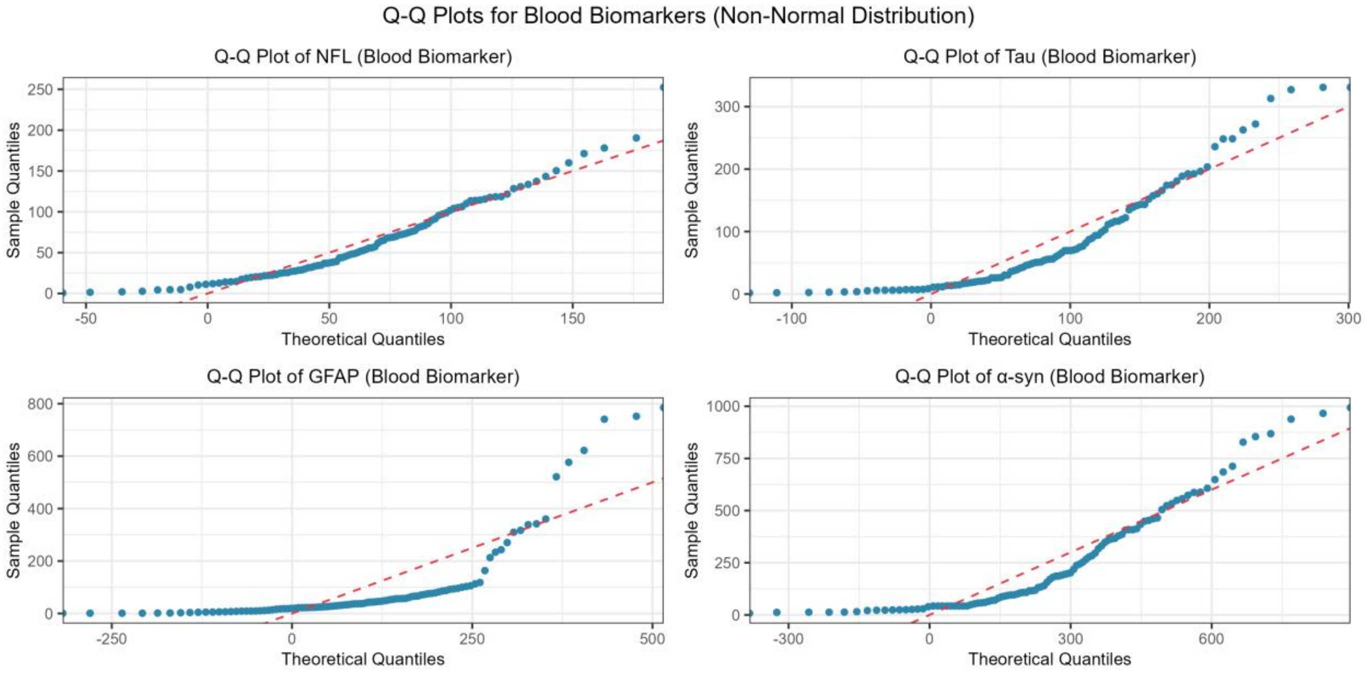

Table S1：The demographic characteristics, clinical information, and plasma biomarker levels of each group

Variable	HC (N=56)	PD (N=54)	MSA (N=25)	PSP (N=10)	CBD(N=5)	*P-value*
**Demographics**						
Age, years	66.0 [60.8, 71.3]	67.5 [55.3, 74.0]	69.0 [60.0, 75.0]	69.5 [57.5, 72.5]	80.0 [74.0, 82.0]	0.384
Gender（Female）	29 (52%)	28 (52%)	13 (52 %)	5 (50 %)	4 (80 %)	0.937
Education, years	9.00 [6.00, 12.0]	6.00 [6.00, 9.00]	6.00 [6.00, 12.0]	6.00 [6.00, 15.0]	6.00 [6.00, 6.00]	0.42
**Plasma Biomarkers (pg/ml)**						
NFL	37.5 [22.7, 78.1]	50.0 [25.0, 88.4]	57.4 [21.9, 114]	141 [89.3, 171]	63.6 [52.7, 98.7]	0.003*
Tau	19.8 [7.07, 49.5]	88.3 [40.0, 172]	121 [60.4, 160]	97.1 [70.1, 143]	69.6 [43.0, 113]	<0.001*
GFAP	22.1 [5.85, 41.4]	47.1 [23.2, 86.3]	86.4 [31.8, 343]	85.7 [41.9, 445]	120 [66.4, 338]	<0.001*
α-Synuclein	42.5 [24.1, 62.4]	288 [170, 508]	378 [238, 523]	388 [335, 466]	367 [252, 587]	<0.001*
**Clinical Characteristics**						
Disease Duration, years	NA	5.00 [2.00, 8.00]	2.00 [1.00, 3.00]	4.50 [1.00, 5.75]	2.00 [2.00, 3.00]	0.002*
LEDD, mg/day	NA	560 [375, 664]	238 [100, 574]	335 [300, 441]	550 [463, 700]	0.02*
Hoehn & Yahr Stage	NA	3.00 [2.00, 3.00]	3.00 [2.50, 4.00]	3.00 [2.25, 3.00]	3.00 [3.00, 4.00]	0.293
UPDRS III	NA	32.5 [24.3, 37.0]	42.0 [30.0, 47.0]	33.5 [28.8, 39.3]	39.0 [38.0, 47.0]	0.066
MMSE	26.0 [24.0, 27.0]	24.0 [21.0, 27.8]	24.0 [19.0, 28.0]	23.5 [19.5, 25.8]	24.0 [8.00, 28.0]	0.512
PSQI	10.0 [7.75, 12.0]	10.0 [4.50, 13.0]	11.0 [5.00, 12.0]	6.00 [5.00, 10.8]	5.00 [0, 11.0]	0.372
HAMA	7.00 [6.00, 9.00]	10.5 [5.00, 13.0]	9.00 [6.00, 13.0]	8.00 [4.25, 10.0]	10.0 [10.0, 12.0]	0.197
HAMD	8.00 [7.00, 12.0]	9.00 [6.25, 13.0]	7.00 [5.00, 11.0]	6.50 [4.25, 12.3]	7.00 [6.00, 8.00]	0.523

Abbreviations: NFL = Neurofilament Light Chain, GFAP = Glial Fibrillary Acidic Protein, Tau = Microtubule-associated protein tau, LEDD = Levodopa Equivalent Daily Dose, MMSE = Mini-Mental State Examination, UPDRS III = Unified Parkinson's Disease Rating Scale Part III, PSQI = Pittsburgh Sleep Quality Index, HAMA = Hamilton Anxiety Rating Scale, HAMD = Hamilton Depression Rating Scale. * p <0 .05; p-value is the result of Kruskal–Wallis test.

Figure S2(A-F)   ROC Curves for Comparisons Between APS Subgroups and PD

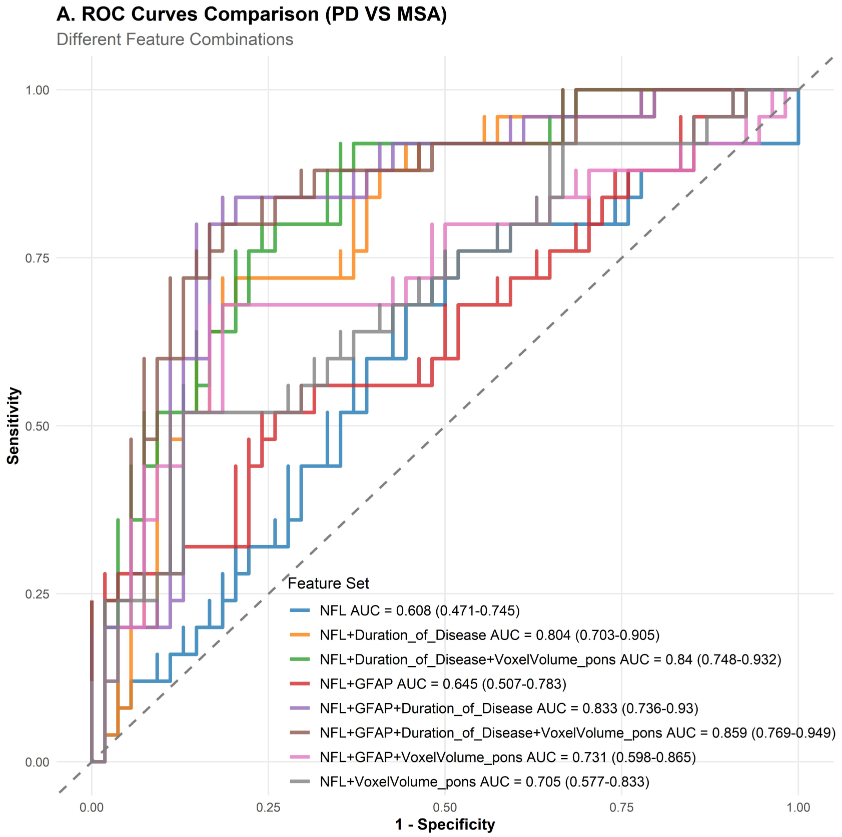

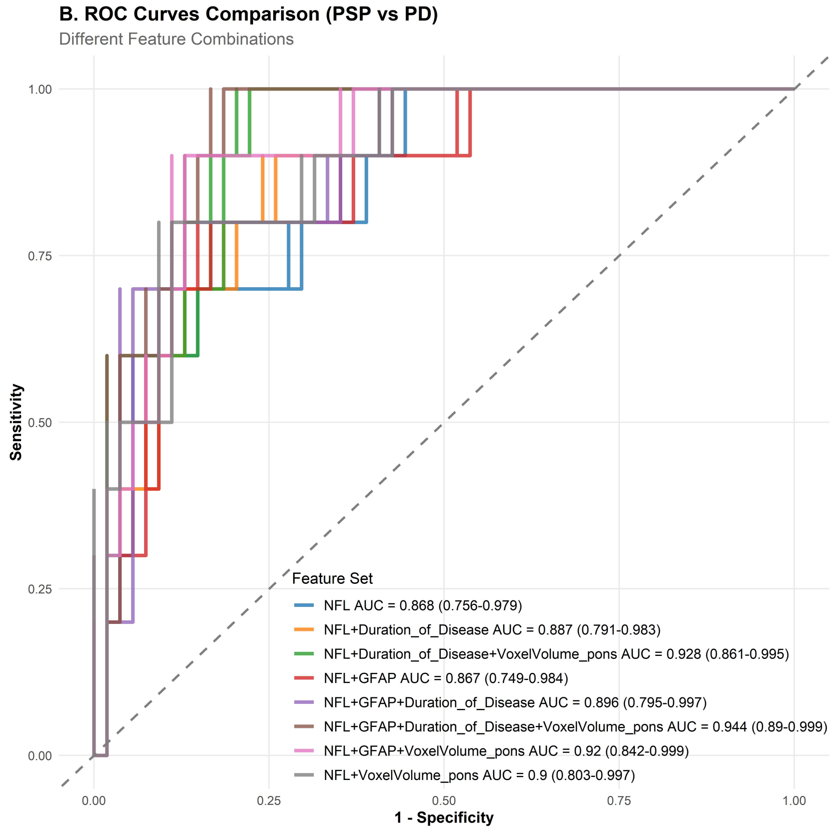

C. ROC Curves Comparison (CBD vs PD)

Different Feature Combinations

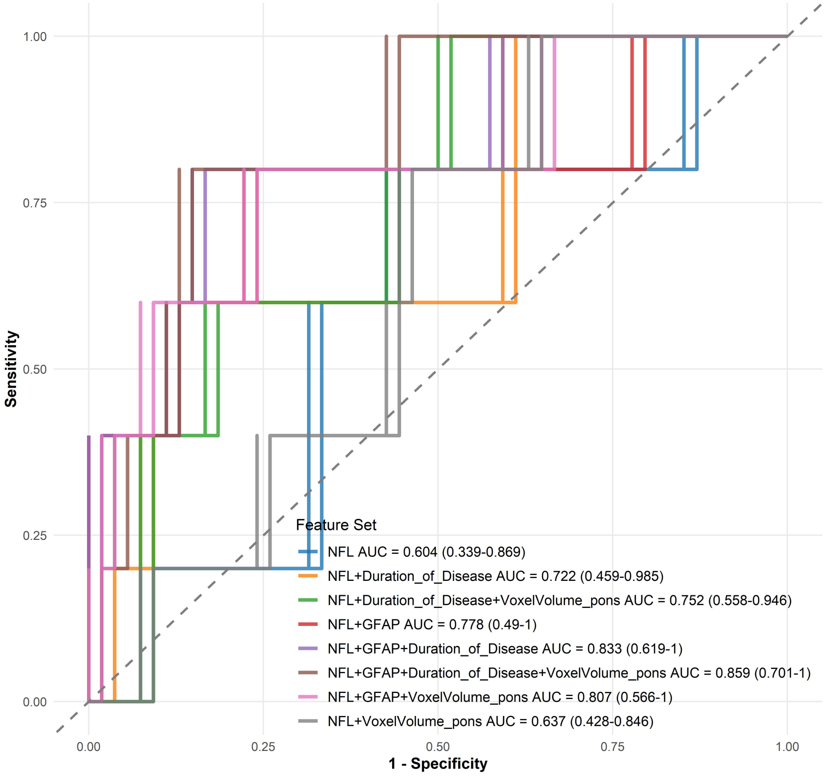

D. ROC Curves Comparison (CBD vs MSA)

Different Feature Combinations

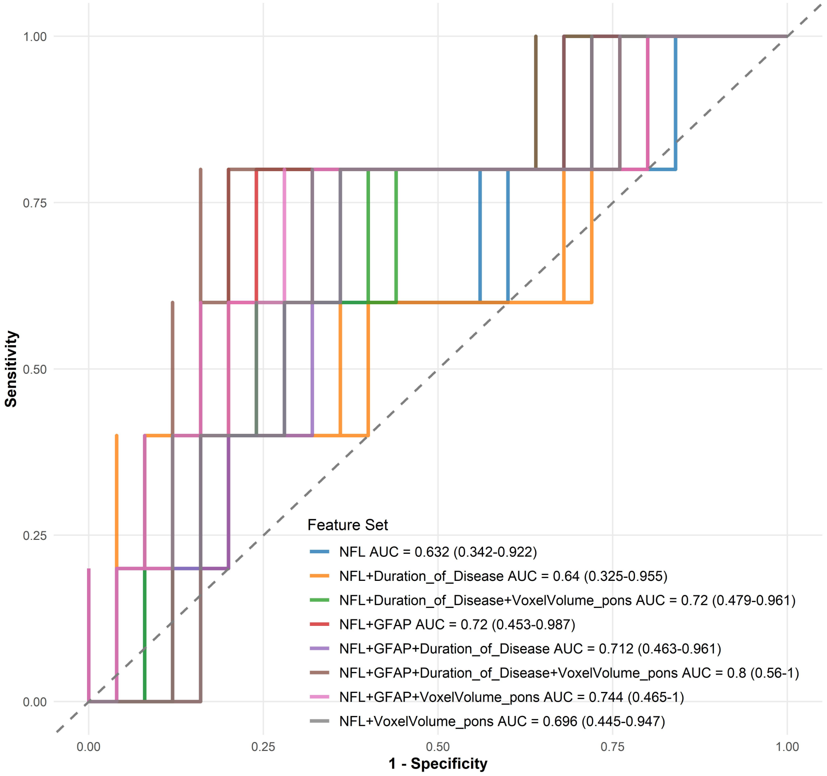

E. ROC Curves Comparison (PSP vs MSA)

Different Feature Combinations

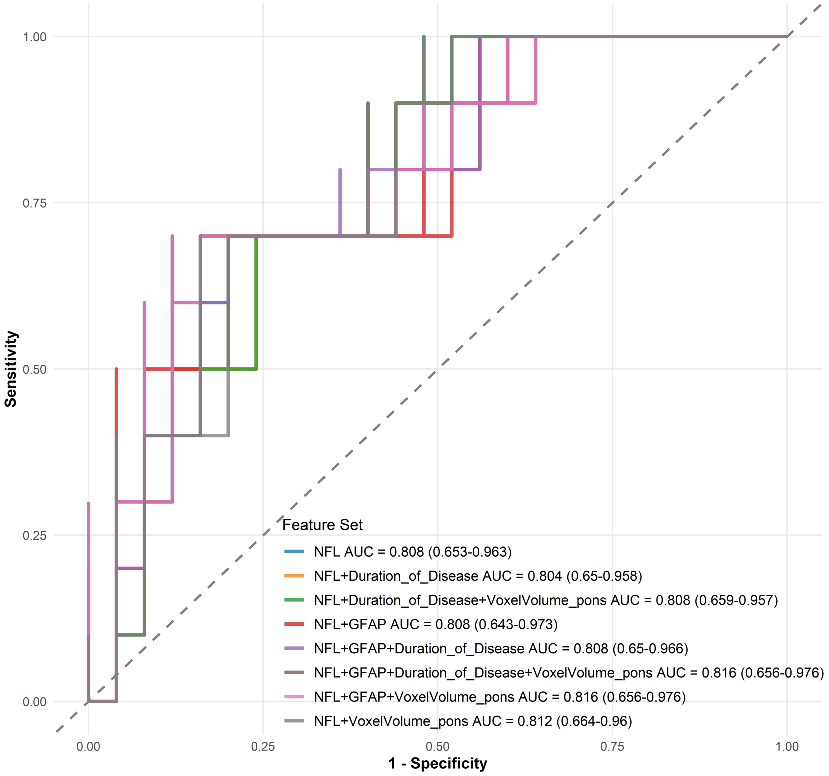

F. ROC Curves Comparison (CBD vs PSP)

Different Feature Combinations

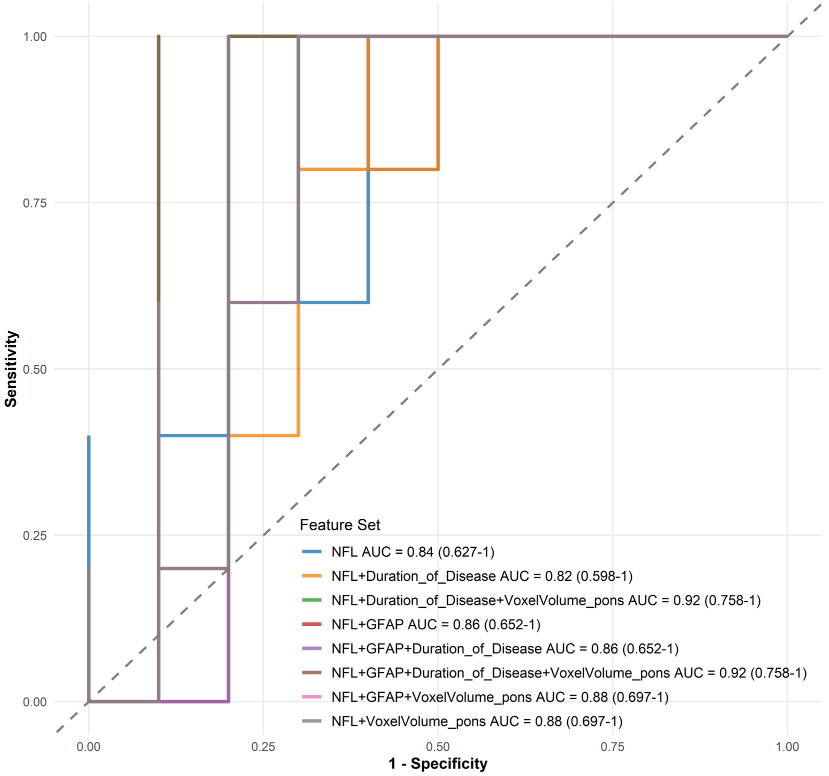

Supplement: Supplementary file 1 [file Data_Sheet_1.pdf]
